# Trans-anal endoscopic microsurgery for internal rectal prolapse

**DOI:** 10.1007/s10151-015-1412-4

**Published:** 2015-12-21

**Authors:** A. L. A. Bloemendaal, M. De Schepper, A. Mishra, R. Hompes, O. M. Jones, I. Lindsey, C. Cunningham

**Affiliations:** Department of Colorectal Surgery, Churchill Hospital, Oxford University Hospitals NHS Trust, Old Road, Headington, Oxford, OX3 7LE UK

**Keywords:** Trans-anal endoscopic microsurgery, Internal rectal prolapse, Trans-anal endoscopic rectal prolapse procedure

## Abstract

Internal rectal prolapse can lead to obstructed defecation, faecal incontinence and pain. In treatment of frail or technically difficult patients, a perineal approach is often used, such as a Delorme’s or a STARR. However, in case of very high take-off prolapse, these procedures are challenging if not unsuitable. We present trans-anal endoscopic microsurgery as surgical option for management of this uncommon type of rectal prolapse in specific cases.

## Introduction

Rectal prolapse can be a disabling disorder, often leading to pain, incontinence and rectal bleeding [1]. Internal rectal prolapse (IRP) is a less obvious disorder, which may lead to obstructive defaecation, faecal incontinence, urge and pain [2].

Both ERP and IRP can be managed by perineal and trans-abdominal approaches. In Europe, the most performed abdominal procedure is the laparoscopic ventral mesh rectopexy [3]. The two most important per-anal procedures are the Delorme’s procedure and the stapled trans-anal rectum resection (STARR) procedure [4]. In rare cases, however, both STARR and internal Delorme’s procedure are limited in reaching the apex of the prolapse in the case of very high take-off.

This paper will discuss using a trans-anal endoscopic microsurgery (TEM) procedure to deal with symptomatic very high take-off internal rectal prolapse, a technique we call the “TransAnal Endoscopic (internal) Rectal Prolapse” procedure, the TERP procedure.

## Technique

### Workup

The preoperative workup should consist of at least a defecating proctogram (or dynamic MRI), showing a high take-off internal rectal prolapse. In the presented cases, patients had undergone multiple investigations prior to surgery. To assess the exact size and nature of the prolapse, we advise an examination under anaesthetic. We use a circular anal dilator (CAD) (Frankenman International Ltd, Hong Kong, China) to visualize the rectum and, using a Babcock, we exert careful traction on the prolapse to assess size, mural thickness and location. In the presented cases, a full-thickness prolapse was seen.

### Operation

**Fig. 1:**
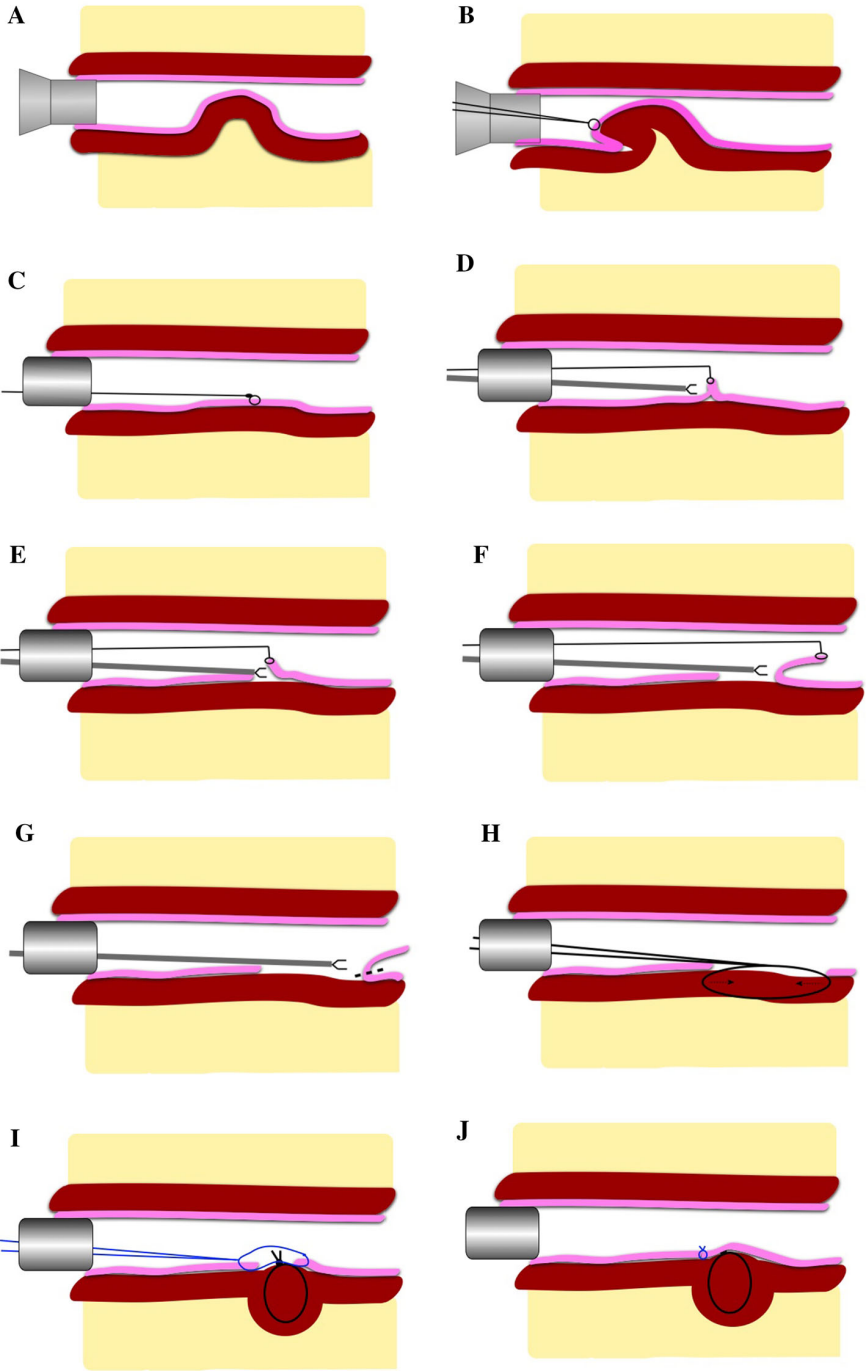
TEM procedure adapted for treatment of internal rectal prolapse (rectal intussusception) and as described in text, the TERP procedure. The mucosa is depicted in *pink* and the muscular tube in *brown*. The mesorectum is depicted in *yellow*. **a** CAD inserted to assess prolapse. **b** Suture placed on apex of prolapse. **c** TEM tube placed and pneumorectum achieved. **d** Using suture for traction, mucosa is dissected (if necessary full-thickness resection can be performed). **e** + **f** Further dissection of mucosa. **g** Transection of mucosa (*dotted line*). **h** Plication sutures to muscular layers. **i**: Plication of muscular layer (**i** + **j**)

**Fig. 2:**
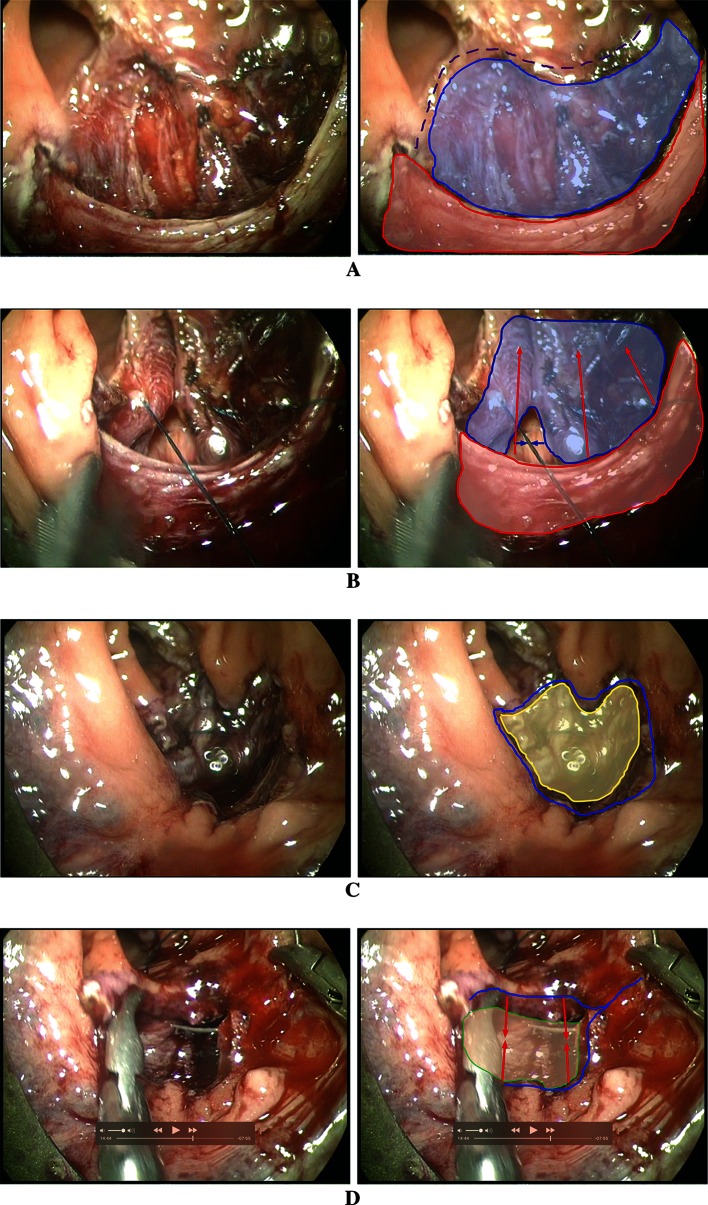
Stills from TERP procedure for IRP with solitary rectal ulcer. **a** Defect after (sub)mucosal resection. In presented case, a partial full-thickness resection was performed (*blue area*) due to inflammatory destruction of planes by rectal ulcer. The longitudinal muscular fibres are clearly seen. *Red area* depicts the circular muscular layer. *Purple dotted line* shows the proximal mucosal border. **b** Closure of the full-thickness defect (*blue area*). *Red area* shows the circular muscular layer, and *arrows* depict direction of plication. c After muscular plication: muscular layer in *yellow*. *Blue line* depicts mucosal defect to be closed over plicated muscles. **d** Mucosal closure. *Green area* shows plicated muscles below. *Blue line* depicts mucosal border to be closed

The procedures (Figs. 1, 2) were performed under general anaesthesia in Lloyd-Davies position (the presented cases were posterior prolapses). Intravenous antibiotic prophylaxes were administered (gentamicin 120 mg/metronidazole 500 mg), and an indwelling urinary catheter was placed. An anal block was performed using 20 cc of 0.5 % bupivacaine. A circular anal dilator (CAD) (Frankenman International Ltd, Hong Kong, China) was used to visualize the prolapse and marked at the apex with (a) suture(s) (Fig. 1a + b). The lateral, proximal and distal aspects of the prolapse are marked with diathermy to help determine the size of the resection after pneumorectum is established. Marking the proximal aspect is hampered by poor visualization. The TEM tube (Richard Wolf, Knittlingen, Germany) was inserted and secured (Fig. 1c). In the presented cases, a long tube (200 × 40 mm) was necessary to reach the high take-off prolapse. Pneumorectum was achieved at a pressure of 12 mmHg. Using the suture(s) for outward traction (by lifting with a grasper), a mucosectomy was made (Fig. 1d) at the most distal edge of the prolapse. Working from distal to proximal mucosectomy was performed with Harmonic Ace (Fig. 1e–g) (Ethicon Endo-Surgery, Cincinnati, USA). A submucosal excision is performed where possible. However, if scar tissue is present, this hampers the dissection and subsequent plication; therefore, it should be removed full thickness. A full-thickness muscular defect should be closed using a monofilament absorbable suture (Covidien, New Haven, Connecticut, USA). In the case of submucosal excision, the intact muscularis propria should be plicated with interrupted sutures at approximately 10-mm intervals (Fig. 1h, i) (comparable to a Delorme’s procedure) using a PDS 3-0 (Ethicon, Cincinnati, Ohio, USA). Finally, the mucosa is closed using a V-lock suture (Fig. 1j) (Covidien, New Haven, Connecticut, USA). A rectal catheter is placed, and both rectal and urinary catheters are removed the first postoperative day. Patients are allowed to eat and mobilize freely the first postoperative day.

## Case reports

### Patient A

A 37-year-old female with type III Ehlers–Danlos syndrome was seen in another centre with severe symptoms of ODS and high-grade internal prolapse. She was treated with a rectopexy, followed by a sigmoid resection and a reduction. A cystoplasty was performed for a dysfunctional bladder. The ODS recurred and a STARR procedure followed. Ten months later, symptoms returned and she was referred to our centre. Investigations demonstrated an internal rectal prolapse with high take-off. This prolapse could not be reached by STARR or by perineal approach. The only option was a tailored internal Delorme’s procedure by TEM (TERP procedure), which was undertaken.

She made a good early postoperative recovery, but suffered a secondary haemorrhage at 12 days, which was successfully managed without intervention as an outpatient. After 1 year, the symptoms returned and EUA showed an anterior recurrence of IRP and an anterior rectal ulcer. CT colonography showed a redundant loop of transverse colon adjacent to the pelvic anastomosis of the previous sigmoid resection. For this reason, a left hemicolectomy was performed, resulting in a marked improvement to date.

### Patient B

This 39-year-old patient had a long-standing history of obstructive defecation disorder and was treated mainly conservatively. A proctogram showed intra-anal intussusception and a large enterocoele. Colonic transit was normal. Anorectal physiology showed results within normal range. She underwent a laparoscopic ventral mesh rectopexy in October 2005. Results were poor. Sacral nerve stimulation was tried, but unsuccessful. In the following years, a number of procedures were performed: sigmoid resection, Botox injected into the pelvic floor musculature, refashioning of the LVMR, an anterior Delorme’s procedure and a STARR. A loop ileostomy was formed in 2008 and closed 3 months later. Another STARR followed in 2010. Another EUA showed a very high take-off posterior full-thickness prolapse, beyond reach of normal perineal procedures for rectal intussusception. A TERP procedure followed in October 2011. The results were not completely satisfactory, and a posterior mesh rectopexy was performed in October 2012, which resulted in a definitive improvement of the ODS and no further treatment to date.

### Patient C

This 72-year-old patient, suffering obstructive defecation and incomplete emptying for many years, attended our Colorectal Surgery Clinic in November 2014 regarding a lesion at the anterior anorectal junction suspicious for malignancy at sigmoidoscopy. Pathology showed chronic inflammation and no dysplasia or malignancy. A TERP procedure was performed in January 2015 to resect the lesion in toto. During this procedure, a large anterior rectal prolapse was seen. Pathology of the excised tissue at TERP confirmed inflammation, showing a solitary rectal ulcer. On follow-up in June 2015, the patient noted to have a remarkable improvement in his bowel functions, opening his bowels daily without the sensation of incomplete emptying.

## Discussion

Laparoscopic rectopexy is generally the first-line elective treatment for rectal prolapse. Perineal and trans-anal approaches are more commonly used in patients who cannot undergo abdominal surgery, either due to frailty, or due to technical reasons such as previous laparotomies. In some cases, the take-off and apex of the IRP may be too proximal to be reached by STARR or Delorme’s. In these situations, an adapted TEM procedure, the TERP procedure as described in our case reports, can be performed to resect the prolapse and perform an anastomosis in view. TEM is a minimally invasive technique and has no deleterious long-term effect on anorectal function [5–7].

It is interesting to note that in an early publication from professor Buess’ group exploring the functional impact of TEM surgery, a reduction in perineal descent was noted on defecating proctography after TEM surgery [5]. This improvement has been postulated to be due to either perirectal fibrosis or a consequence of excision of rectal mucosa. The clinical significance of such a finding may be important in the potential development of TEM in rectal prolapse surgery.

In our experience, TERP is a safe and effective procedure for very proximal internal rectal prolapse and our follow-up results have been satisfactory, albeit in a limited number of complex cases.
